# The Landscape of Glucose-Lowering Therapy and Cardiovascular Outcomes: From Barren Land to Metropolis

**DOI:** 10.1155/2017/9257930

**Published:** 2017-11-14

**Authors:** Mona P. Nasrallah, Charbel Abi Khalil, Marwan M. Refaat

**Affiliations:** ^1^Division of Endocrinology and Metabolism, Department of Internal Medicine, Faculty of Medicine, American University of Beirut, Beirut, Lebanon; ^2^Department of Genetic Medicine, Weill Cornell Medical College, Ar-Rayyan, Qatar; ^3^Division of Cardiovascular Medicine, Department of Internal Medicine and Department of Biochemistry and Molecular Genetics, Faculty of Medicine, American University of Beirut, Beirut, Lebanon

## Abstract

The choice of glucose-lowering therapy (GLT) has expanded to include 11 different classes in addition to insulin. Since the 2008 Food and Drug Administration guidance for industry and mandate of demonstrating cardiovascular (CV) safety prior to any new drug approval, there were several trials primarily conducted to establish that goal. Some had neutral effects, while there were positively beneficial outcomes with more recent studies. Hospitalization for congestive heart failure has also been a heterogeneous finding among the different classes of GLT, with drug outcomes ranging from risky to beneficial. The current review selectively focuses on the evidence for CV outcomes for each class of GLT and summarizes the existing guidelines with regard to these drugs in heart disease. Moreover, it illustrates the dynamic status in the development of evidence. Finally, the review enables healthcare providers to formulate a plan for hypoglycemic therapy which will optimize CV health, in a patient-centered manner.

## 1. Introduction

*Case*. A 58-year-old man presents to the endocrine clinic for Type 2 Diabetes (T2D) management. He was diagnosed with T2D 8 years priorly, after hospitalization for acute myocardial infarction (MI), for which he underwent urgent revascularization with a stent placed in the left anterior descending artery. Since then, he has been on medical therapy and lifestyle management. He stopped smoking, decreased his Body Mass Index (BMI) from 32 to 29.5 kg/m^2^, and started exercise two to three times per week. His work is stressful as a regional manager and involves frequent travel. He has no other significant comorbidities. His T2D medication is metformin 2000 mg per day. In addition, he is on antiplatelet therapy, angiotensin receptor blockade, beta blockade, and high intensity statin.

His studies reveal hemoglobin A1c (HbA1c) 8.3%; ALT 45 IU/L; creatinine 0.9 mg/dL with eGFR > 60 mL/min/1.73 m^2^, potassium 4.5 mmol/L, glucose 135 mg/dL, LDL-C 62 mg/dL, HDL-C 42 g/dL, triglyceride 180 mg/dL, total cholesterol 140 mg/dL, hemoglobin of 13 g/dL with MCV of 92 fl, and urine microalbumin/creatinine ratio of 40 mg/g. Echocardiogram done one year ago shows moderate to good ejection fraction of 55% with mild apical hypokinesia.

The management of this patient falls under the American Diabetes Association (ADA) guidelines for comprehensive diabetes care, in terms of lifestyle recommendations and preemptive measures such as immunization updates, dental checks, cancer screening, and complications prevention [1]. However, the specific management of his hyperglycemia raises the question on how to improve his metabolic control in a manner to optimize cardiovascular (CV) health.

Due to the rapid accumulation of knowledge in the field of glycemic control and CV outcomes, there have been a number of reviews shedding different perspectives on this topic, within the past two years [2–8]. The current paper comprehensively assesses the existing classes of glucose-lowering therapy (GLT) with analysis of the available CV data, so that the clinician can make informed patient-centered recommendations, supported by an updated body of evidence.

## 2. Historical Overview of Diabetes Therapy and Cardiovascular Effects 

The pharmacologic therapy for T2D began in 1921 with the landmark discovery of insulin, followed by the availability of sulfonylureas (SU) and biguanides in the 1950s [9]. Guidelines for T2D therapy were largely shaped by the United Kingdom Prospective Diabetes Study (UKPDS), which has demonstrated that targeting an HbA1c of 7.0% as compared to 7.9%, in subjects newly diagnosed with T2D, reduced microvascular complications by 25% [10]. Macrovascular endpoints were more difficult to establish; in the strict glycemic control group, only the subgroup of overweight subjects on metformin had a significantly lower rate of CV events and mortality [11]. Because of the rapid growth of the epidemic and the multiplicity of pathophysiologic mechanisms in T2D, the development of new classes of drugs continued in an accelerated manner [9].

Prior to 2008 and largely guided by the UKPDS findings during the preceding decade, the FDA requirements for approval of a new hypoglycemic agent relied on demonstration of efficacy in glucose-lowering using HbA1c as a surrogate marker of vascular endpoints, granted no major adverse effects of the drug were observed in preclinical and clinical studies [12, 13]. The trials were typically short in duration and tended to exclude subjects with preexisting CV disease or renal insufficiency [13]. However, the vast majority of morbidity and mortality in T2D is a result of CV complications. Furthermore, diabetes drugs are typically consumed for many years, and any untoward late CV effect of a drug would likely be missed in these phase 3 clinical trials. One such example was the suspicion that was raised in 2007 regarding rosiglitazone and increased CV events [14]. Rosiglitazone, approved in 1999, was the most widely prescribed hypoglycemic agent at the time of the controversy due to its promising insulin-sensitizing profile. More clinical evidence on rosiglitazone is described under Thiazolidinediones. One additional unexpected finding which occurred during the same time period as the rosiglitazone controversy was the outcome of three large trials which showed that intensive glycemic control either provided no macrovascular benefit [15] or was associated with increased mortality [16]. Therefore, the use of HbA1c was no longer a valid intermediate marker of macrovascular outcomes.

All of the above factors have led the FDA to mandate evidence of CV safety prior to approval of any new potential GLT. Specifically, the drug had to demonstrate noninferiority in CV outcomes, with an upper bound hazard ratio of 1.3 at 95% confidence interval (CI), in order to be approved. Alternatively, the drug could show a noninferiority HR = 1.8 with conditional approval pending demonstration of CV safety at 1.3 in postmarketing studies [17]. As a result, trials testing new hypoglycemic therapies after December 2008 have tended to be much more homogenous and comparable in nature: subjects included had more CV risk factors including renal insufficiency, and trial duration was longer; the drug was tested against placebo on a background of “standard of care” T2D therapy; finally in the phase 3 testing of most drugs, the trial had as primary outcome major CV endpoints, which were independently adjudicated.

## 3. Available Hypoglycemic Classes

Along with insulin, there are currently 11 different classes of FDA-approved GLTs. The body of evidence on CV safety for drugs which became available after 2008 tends to be more robust, as a result of the changed FDA requirements. Nonetheless, data does exist for most other classes and will be reviewed below.

### 3.1. Sulfonylureas (SUs)

SUs are blockers of the ATP-sensitive potassium channel of the beta islet cell and as such promote secretion of insulin [8]. The main risk of SUs is hypoglycemia and weight gain, especially when aiming for tight glucose control. They may also be associated with more rapid beta cell failure [18].

The earliest prospective, randomized double-blinded study to report on CV effects in T2D therapy is the University Group Diabetes Program conducted on 823 subjects assigned to the first-generation SU tolbutamide, lifestyle, insulin, or phenformin [19]. There was increased mortality in the tolbutamide group, and even though the study was criticized for not being powered enough, first-generation SUs were replaced by newer agents which cause less hypoglycemia. The second large body of evidence came from the 10-year follow-up of the UKPDS, whereby subjects initially randomized to the intensive glucose arm and who received the SUs chlorpropamide, glibenclamide, or glipizide had reduction of 15% in MI and 13% in overall mortality compared to the conventional group [20]. The latter was achieved despite the HbA1c becoming similar in both groups after trial completion and averaging 7.8% during the 10-year follow-up. While the target HbA1c of less than 7% in UKPDS was beneficial, the Action to Control Cardiovascular Risk in Diabetes (ACCORD) trial cast doubt as to the possibility of achieving tighter glycemic control of HbA1c less than 6.5% safely [16]. In this study conducted on 10250 adults with T2D, the group on intensive glycemic control using predominantly insulin, thiazolidinedione, or glimepiride had higher mortality than the conventional group, despite achieving an HbA1c of 6.4% versus 7.5%, respectively [16]. It is important to note that the mortality could not be linked to any single drug class, despite the higher use of thiazolidinediones, secretagogues, and insulin, in the intensive arm. Rather, subjects in the ACCORD trial were on average 10 years older (mean age 62 years) with a mean duration of diabetes of 10 years compared to the UKPDS subjects who were newly diagnosed with T2D. Therefore, the conclusion from that trial was mainly that tighter control in advanced diabetes using classes which predispose to hypoglycemia and weight gain may be deleterious, on the macrovascular level. However, some concerns were appeased when a similar population as that of the ACCORD was tested in the pharmaceutically initiated Action in Diabetes and Vascular Disease: Preterax and Diamicron MR Controlled Evaluation (ADVANCE) trial. In this study, 11140 adults with T2D (mean age of 66 years and mean duration of disease of 8 years) were randomly assigned to either intensive glucose control with gliclazide or standard care for 5 years. By trial completion, the group on gliclazide had HbA1c of 6.5% versus 7.3% in the standard group and also had a lower primary composite outcome, mainly driven by reduction in nephropathy [21]. The benefits were sustained in a 5-year follow-up after the trial finish [22]. Most recently, a meta-analysis which included 47 randomized trials using second- or third-generation SUs against placebo or as add-on to metformin revealed no increase in CV risk [23]. However, the trials were not primarily designed to assess CV outcomes, and the study did not extract information on duration of disease nor presence of underlying CV risk, which constitutes a major limitation to generalizing these results.

### 3.2. Biguanides

Available since 1972, but FDA approved since 1994, metformin is the only existing compound in the class and has stood the test of time with continued benefits described. Metformin's main glucose-lowering effect is through reduction of hepatic gluconeogenesis. In addition, more mechanisms have been described on other parts of the gastrointestinal tract such as increased intestinal glucose utilization, increased glucagonlike peptide-1 levels, altered bile acids, and altered microbiome [24].

The CV benefit of metformin was first demonstrated in UKPDS where the overweight group on intensive glucose control had 39% reduction in MI rate [10]. In addition, in the 10-year follow-up of UKPDS, the long-term benefit for all subjects in the intensive arm on metformin was shown by reducing MI by 33% and all-cause mortality by 27% as compared to conventional control [20].

### 3.3. Alpha-Glucosidase Inhibitors (AGis)

As the name implies, compounds available in this class—acarbose (FDA 1995), miglitol (FDA 1996), and voglibose (developed in Japan and available since 1994)—inhibit the enzyme responsible for breakdown of oligosaccharides into disaccharides at the intestinal brush border [8]. They do not cause hypoglycemia and target postprandial glucose. The main side-effect is gastrointestinal intolerance.

Acarbose has one completed placebo-controlled, randomized trial which assesses progression to T2D and the development of major CV events. In the STOP Non-Insulin Dependent Diabetes Mellitus (STOP-NIDDM) trial, with 1429 adults with impaired glucose tolerance and high CV risk, acarbose given over a mean of 3.3 years significantly reduced acute MI rate as well as the composite of macrovascular events with a drop of 49% relative risk (HR = 0.51; 95% CI: 0.28–0.95) and an absolute risk reduction of 2.5% [25]. Of note is the 24% dropout rate twice as high in the acarbose arm because of gastrointestinal intolerance. Voglibose was evaluated in a placebo-controlled, randomized trial on 859 adults with impaired glucose tolerance and recent acute MI in Japan. The trial ABC (Alpha-glucosidase-inhibitor Blocks Cardiac Events in Patients with Myocardial Infarction and Impaired Glucose Tolerance) was terminated after a two-year period due to total lack of difference between the 2 groups, in terms of CV outcomes. Of note is that the dropout rate was only 3%, as opposed to the large rate seen with STOP-NIDDM trial [26].

Further evidence is expected from the ongoing Acarbose Cardiovascular Evaluation (ACE) trial. This study will assess CV outcomes in more than 6000 adults over the age of 50 years with CV disease and impaired glucose tolerance, on acarbose versus placebo for a mean of 4 years. The primary results are expected to be announced in the fall of 2017 [27].

### 3.4. Meglitinides

Meglitinides are secretagogues which bind the same ATP-dependent potassium channel as SUs, but with shorter onset and duration of action [8]. They are metabolized and, as such, cause less hypoglycemia than SUs, especially in renal disease.

There are two agents available for use, repaglinide and nateglinide, FDA approved since 1997 and 2001, respectively. Studies assessing meglitinides and CV effects are limited. Because these agents target postprandial hyperglycemia, their cardioprotective effect may be similar to other compounds which reduce postmeal glucose excursions such as acarbose in the STOP-NIDDM trial. Nateglinide was used in the Long-Term Study of Nateglinide plus Valsartan to Prevent or Delay Type II Diabetes Mellitus and Cardiovascular Complications (NAVIGATOR) a placebo-controlled trial of 9306 subjects at high CV risk and with impaired glucose tolerance. After a median of 5 years, there was no reduction in the incidence of T2D nor in the composite outcome of CV disease. There were, however, more hypoglycaemia cases in the nateglinide arm [28].

### 3.5. Thiazolidinediones

Thiazolidinediones (TZDs) are insulin sensitizers which have ubiquitous effects on the liver, skeletal muscle, and adipose tissue through peroxisome proliferator-activated receptor gamma binding [29]. Known side-effects are fluid retention, weight gain, anemia, fractures, and exacerbation of heart failure [8]. Two compounds—rosiglitazone (FDA 1999) and pioglitazone (FDA 2001)—are available. A warning regarding pioglitazone and bladder cancer was issued in 2010, and the drug should not be prescribed in case of unexplained hematuria or active bladder cancer [30]. Both agents are contraindicated in subjects at risk of heart failure.

CV concern was raised with rosiglitazone 8 years after marketing, when a meta-analysis of 42 trials showed a 43% increase in the risk of MI (86 versus 72 events) and a 64% increase in death from CV disease (39 versus 22 deaths), even though the latter did not reach significance [14]. The study was criticized for not reporting absolute risk which was only 0.2% higher with rosiglitazone and, more importantly, for having excluded trials which showed no events, that is, 4 studies from the infarction analysis and 19 from the mortality analysis [31]. Nonetheless, rosiglitazone came under scrutiny and its use was heavily restricted by the FDA until the results of the trial designed primarily to assess CV risk came out. The Rosiglitazone Evaluated for Cardiovascular Outcomes in oRal agent combination therapy for type 2 Diabetes (RECORD) trial is an open-label study of 4447 subjects randomized to rosiglitazone versus a comparator group of either metformin or SU, for a mean of 5.5 years. The inclusion criteria did not include high CV risk, and the event rate was relatively lower in both groups for MI (68 versus 60), for all-cause mortality (88 versus 96), and for stroke (50 versus 63), in rosiglitazone versus metformin/SU, respectively. There was no difference in the composite or individual endpoints [32]. There were higher rates of fatal and nonfatal heart failure (61 versus 29 subjects). The results of this pharmaceutical-initiated trial were further reaffirmed by independent review of adjudication [33] and the prescribing restriction was lifted in 2013. However, the trial was limited by a relatively high dropout rate of 18% and by the lack of blinding [34]. Rosiglitazone use remains limited among physicians.

Pioglitazone, the other compound available in this class, was also scrutinized when concerns about rosiglitazone were raised. However, a trial with primary CV endpoints was reassuring: the PROspective PioglitAzone Clinical Trial in macroVascular Events (PROactive) enrolled 5238 patients with T2D and established macrovascular disease, randomized to pioglitazone versus placebo, in addition to standard of care. Strangely, the trial was terminated early, after a 3-year follow-up, even though there was no difference in the primary 7-point composite outcome. However, a secondary outcome of nonfatal MI, stroke, or death was lower in the pioglitazone arm with 301 out of 2605 versus 358 out of 2633, for pioglitazone and placebo, respectively [35]. The authors were criticized for reporting a nonpredefined secondary outcome when the primary was negative [36]. Nonetheless, the reduction was consistent across all 3 components of the 3-point major adverse CV events (MACE). In line with these findings was a meta-analysis of 19 trials on pioglitazone which showed a risk reduction in MI, stroke, or death by 18% (HR = 0.82; 95% confidence interval [CI], 0.72–0.94; *p* = 0.005). The authors note that data from PROactive trial constitutes the bulk of events; nonetheless, the results from other trials in the meta-analysis were consistent [37]. As expected, there was a higher rate of heart failure (16% versus 11.5%); however, it did not result in increased mortality.

### 3.6. Amylin Analogues or Amylinomimetics

Pramlintide (FDA 2005) is an incretin cosecreted with insulin, which suppresses glucagon and delays gastric emptying [8]. As such, it targets postprandial glucose and does not induce hypoglycemia if used as monotherapy. However, its use is recommended as add-on to insulin, in which case cautious glucose monitoring is necessary to prevent severe hypoglycemia. Its mechanism of action (targeting postprandial glucose) would suggest favorable CV outcome; however no studies exist. Its use has been limited by twice-daily injections, relatively high cost, and guidelines which narrow its use to a mere add-on to prandial insulin [8].

### 3.7. Bile Acid Sequestrants

Colesevelam is the only hypoglycemic agent approved for such use in this category because it incidentally lowers HbA1c by 0.5% [8]. It obtained FDA approval, in January 2008, for use in T2D as an adjunct to diet and exercise. Its mechanism of action is unclear but may decrease intestinal glucose absorption. Its main side-effects are gastrointestinal.

The only study reporting on CV outcomes was a retrospective chart review in subjects with T2D and dyslipidemia, comparing those on colesevelam (*n* = 847) to those on ezetimibe (*n* = 3384). After adjustment for any baseline differences, fewer subjects on colesevelam had the primary CV event (HR = 0.58,  *p* = 0.004). However, the authors themselves concluded that change in clinical practice cannot be made based on this study alone due to the limitation of a retrospective design [38].

### 3.8. Dopamine Agonists

An immediate-release formulation of bromocriptine is postulated to restore the circadian peak of dopaminergic activity in the hypothalamus and as such to decrease hepatic gluconeogenesis and insulin resistance [39]. Bromocriptine-QR (FDA 2009) is administered within two hours of waking up. Its main side-effects are nausea, dizziness, and orthostasis.

In a primary CV endpoint placebo-controlled trial, bromocriptine or placebo was administered over 12 months to 3070 subjects with T2D, in addition to standard therapy. A quarter of patients had preexisting CV disease. Bromocriptine reduced the composite outcome which included MI, stroke, revascularization, hospitalization for cardiac cause, and death to 32 versus 37 events (HR = 0.60; 95% CI: 0.37–0.96). However, the study was limited by a large number of subjects stopping the drug prior to final visit: 47% in bromocriptine group and 32% in the placebo group [39].

### 3.9. Dipeptidyl Peptidase-4 Inhibitors (DPP-4is)

DPP-4 inhibitors increase endogenous levels of glucagonlike peptide-1 (GLP-1) and as such act as mild hypoglycemic oral agents. There are currently 4 FDA-approved agents: sitagliptin (FDA approved in 2006), saxagliptin (FDA approved in 2009), linagliptin (FDA approved in 2011), and alogliptin (FDA approved in 2013). Vildagliptin was mandated by the FDA in 2007 to conduct more trials in patients with renal insufficiency; there has been no reapplication for FDA approval since then, but it remains widely used in other parts of the world. Additionally, there are 2 once-weekly DPP-4i—trelagliptin and omarigliptin—both available in Japan. Side-effects from DPP-4 inhibitors have been described in postmarketing studies. The FDA issued a warning about a rare, but real, risk of pancreatitis for all agents in this class. The risk of pancreatic cancer remains a subject of debate [8]. Because the enzyme dipeptidyl peptidase exists in several forms and because DPP-4 activity is specifically exhibited by the cell surface protein CD26 of the T-lymphocyte, this class of drugs has also been associated with various disorders resulting from modulation of immune function such as autoimmune-related skin conditions (notably bullous pemphigoid), arthralgia, myalgia, and nasopharyngitis [40]. There are also agent-specific concerns on heart failure as described below.

CV safety was established for most currently available DPP-4 inhibitors. The first trial with CV endpoints, the Saxagliptin Assessment of Vascular Outcomes in Patients with Diabetes Mellitus- Thrombolysis in Myocardial Infarction 53 (SAVOR-TIMI 53), enrolled 16 492 adults above 40 years of age, who had established CV disease or were at high risk for CV disease, who received saxagliptin or placebo, along with usual care, and who were followed for 2.1 years. At the end of trial, despite a small difference in the HbA1c of 0.2% in the intervention group (7.7 versus 7.9%), there was no difference in the primary endpoint of nonfatal MI, ischemic stroke, or CV death (613 in saxagliptin versus 609 in placebo group, HR = 1.00; 95% CI: 0.89–1.12, *p* < 0.001 noninferiority) [41]. However, there were more subjects who were hospitalized for nonfatal heart failure (289 in saxagliptin versus 228 in placebo group, HR = 1.27; 95% CI: 1.07–1.51, *p* = 0.007). Risk factors for heart failure were prior heart failure, a lower eGFR, and higher baseline pro-BNP levels [42]. Furthermore, the effect of the drug on heart failure was no longer seen one year into the trial.

In the second trial, the Examination of Cardiovascular Outcomes with Alogliptin versus Standard of Care (EXAMINE), 5380 men and women with acute coronary syndrome within the last 15–90 days were randomized to alogliptin or placebo, in addition to standard of care. After a median of 18 months, the difference in HbA1c between groups was only 0.3%, and there was no difference in the primary 3-point MACE (316 events for alogliptin versus 305 for placebo, HR = 0.96; upper boundary CI < 1.16). Hospitalization for heart failure occurred in 85 of alogliptin-treated patients versus 79 in the placebo group; however this number did not reach statistical significance [43].

In the third trial, Trial Evaluating Cardiovascular Outcomes with Sitagliptin (TECOS), 14,671 adults above 50 years of age, with established or at high CV risk, were assigned sitagliptin versus placebo in addition to standard of care [44]. After a median follow-up of 3 years, the HbA1c was 0.29% lower in the sitagliptin group; however, there was no difference in the primary 3-point MACE (839 events for sitagliptin versus 851 for placebo, HR = 0.98; 95% CI: 0.88–1.09, *p* < 0.001 for noninferiority). There were 228 subjects hospitalized for heart failure versus 229, in sitagliptin and placebo groups, respectively. The latter clearly indicates there was no increased risk of heart failure exacerbation, in the case of sitagliptin.

As mentioned, there is no primary CV outcome trial for vildagliptin. However, a meta-analysis which included 69 studies on 28,006 subjects on vildagliptin versus a comparator found no increased risk of CV events or heart failure [45]. In a 12-month Vildagliptin in Ventricular Dysfunction Diabetes (VIVIDD) trial 254 patients with NYHA Classes I–III heart failure were randomized to vildagliptin or placebo. There were 13 admissions for heart failure in the vildagliptin group versus 10 in the placebo group. Although the number did not reach statistical significance, the end-diastolic volume was higher in those on vildagliptin, again reinforcing previous suspicions about the group [46].

A systematic review and meta-analysis on DPP-4 inhibitors found a suggestion of increased heart failure risk, primarily driven by SAVOR, EXAMINE, and VIVIDD trials [47]. One proposed physiologic explanation for the heart failure finding is the inhibitory effect of this class on glucagon, a positive inotropic hormone [48]. One other advanced theory is the inhibition of breakdown of Neuropeptide Y, also a substrate of DPP-4, leading to vasoconstriction [49]. However, given the lack of consistency of the study results, more data will be helpful to further elucidate the question of DPP-4 inhibition and effect on heart failure. The CARdiovascular Outcome study of LINAgliptin versus glimepiride in patients with Type 2 Diabetes (CAROLINA) has randomized 6051 subjects above 40 years of age with either high risk or preexisting CV disease to linagliptin or glimepiride; its results are anticipated in the middle of 2019 [50]. Out of the once-weekly DPP4is, trelagliptin does not have a CV trial linked to it. Omarigliptin was undergoing CV assessment in a trial expected in 2021; however, the trial was terminated by the company MSD earlier than schedule (in 2016), with the announcement that the decision was made for marketing purposes and not for medical reasons [51, 52].

In summary, the use of DPP-4 inhibitors in subjects with CV disease seems neutral in terms of event outcomes. There appears, however, to be a small signal for heart failure, especially in those at risk.

### 3.10. Glucagonlike Peptide-1 Receptor Agonists (GLP-1 RA)

GLP-1 normally secreted by the ileum stimulates insulin release in a glucose-dependent manner, inhibits glucagon release, and suppresses appetite both centrally and by delayed gastric emptying [8]. The class has been available since 2005 with several compounds: exenatide (FDA approved in 2005 for the twice-daily injection, FDA approved in 2012 for the once-weekly one), liraglutide (FDA approved in 2014), dulaglutide (FDA approved in 2014), albiglutide (FDA approved in 2014), lixisenatide (FDA approved in 2016), and semaglutide (application to FDA submitted December 2016). Concerns as a class have mainly been pancreatitis and a common side-effect is nausea. More recently, there are reports about gallbladder disease with increased risk of cholecystitis [53]. They are used with caution in people at risk of medullary thyroid cancer. There are GLP-1 receptors on the heart and questions were raised in view of the DPP-4 inhibitor results on heart failure; there is a mild, but consistent, increase in heart rate with all GLP-1 agonists [48]. This effect may be heterogeneous among the different compounds, with the shortest-acting agent increasing the rate by 1–3 beats per minute, all the way to the once-weekly agents increasing it by 6–10 beats per minute [54]. Despite the concern about heart rate increase and about glucagon inhibition (similar to DPP-4 inhibitors), GLP-1 may also act as a potent inotropic agent off-setting its potential cardiac drawbacks [55].

The first study in class to examine CV risk was the Evaluation of LIXisenatide in Acute coronary syndrome (ELIXA) which randomized 6068 men and women with T2D, who had acute coronary syndrome within the preceding 180 days, to lixisenatide or placebo on a background of usual care. After a median of 25 months, there were 406 events in the lixisenatide group versus 399 in placebo (HR = 1.02, 95% CI: 0.89–1.17, *p* < 0.001 for noninferiority) [56]. There was a similar incidence of hospitalization for heart failure among both groups.

The second outcomes trial, Liraglutide Effect and Action in Diabetes: Evaluation of Cardiovascular Outcome Results (LEADER), randomized 9340 adults above 50 years of age, with established disease or at high CV risk, to liraglutide or placebo against standard of care for a mean of 3.8 years. The average HbA1c at baseline was 8.7% and duration of T2D 12.8 years. By the end of trial, there was only a mild drop in HbA1c in both groups and subjects on liraglutide had a 0.4% lower level [57]. However, there was a significant reduction in the 3-point MACE with 608 events versus 694 in liraglutide and placebo, respectively (HR = 0.87; 95% CI 0.78–0.97, *p* = 0.01 for superiority). The difference was mainly driven by death from CV causes. This started to become apparent after 18 months of exposure to the drug. There was no difference in hospitalization for heart failure. In subgroup analysis, the benefit was observed across all groups. Additionally, there was 22% less risk of nephropathy, which, by itself, represents reduced macrovascular hazard.

A third study in the Trial to Evaluate Cardiovascular and Other Long-Term Outcomes with Semaglutide in Subjects with Type 2 Diabetes (SUSTAIN-6) randomized 3297 subjects to the once-weekly semaglutide at 0.5 or 1.0 mg doses versus placebo. Again, more than 80% of subjects had established CV disease, and the others were at high risk with age above 50 years and duration of T2D of 13.9 years. Baseline HbA1c was 8.7% and the difference at the end of 2.1-year follow-up was 0.7 and 0.9%, for the 0.5 mg and 1.0 mg doses of semaglutide, respectively [58]. The primary outcome of 3-point MACE occurred in 108 on semaglutide and 146 subjects on placebo (HR = 0.74; 95% CI 0.58 to 0.95, *p* = 0.02 for superiority). The outcome started to diverge after an 18-month lag time, and it was mainly driven by nonfatal MI and nonfatal stroke. This remained consistent across subgroup analysis. Similar to the other two GLP-1 trials, there was no difference in heart failure, which occurred in only 3.6 and 3.3%, for semaglutide and placebo, respectively. Also, similar to the liraglutide trial, there was a 36% reduction in the risk of new or worsening nephropathy.

The same investigators, who conducted the latter 2 trials, propose that liraglutide and semaglutide may be effective in reversing or stabilizing atherosclerosis given the lag time to see effects and the consistency of the results across subgroups. In addition, for both liraglutide and semaglutide, there was a small increase in progression of retinopathy and in cholecystitis for liraglutide. No such side-effects were reported in the lixisenatide trial.

There are 3 other once-weekly GLP-1 agonists, with ongoing trials for CV safety. Dulaglutide in the Researching CV Events with a Weekly Incretin in Diabetes (REWIND) and exenatide once weekly in Exenatide Study of Cardiovascular Event Lowering (EXSCEL) trial are both expected at the end of 2018. Finally, albiglutide in the HARMONY OUTCOME trial is expected in 2019 [59].

### 3.11. SGLT2 Inhibitors

Sodium-glucose cotransport 2 (SGLT2) inhibitors partially block glucose reabsorption in the proximal renal tubule by binding to the SGLT2 transporter. Available SGLT2 inhibitors are highly selective for the SGLT2 receptor in the renal tubule. However, there may be minor effect on intestinal SGLT1 inhibition, affecting glucose absorption [8]. Efficacy on HbA1c lowering averages 0.6%. Other than glucose-lowering, SGLT2 inhibitors decrease systolic and diastolic blood pressure mildly. It is preferable not to initiate them if eGFR < 60 mL/min/1.73 mm^2^ (<45 for empagliflozin), and it is recommended to discontinue them if eGFR falls persistently below 45 mL/min/1.73 mm^2^ with their use. They increase the risk of urinary tract infection and genital candidiasis. There are postmarketing reports of euglycemic diabetic ketoacidosis associated with their use. One potential explanation is that SGLT2 transporters are present on the alpha islet cells of the pancreas, and their inhibition results in higher glucagon secretion [48]. In May 2017, the FDA issued a drug safety alert on canagliflozin being associated with twice as much risk of toe and foot amputations, as the placebo group [60]. The mechanism is still unclear; however subjects on canagliflozin tended to have more peripheral vascular disease.

The first agent to be approved by the FDA, canagliflozin, became available in 2013, based on pooled data from 9 studies on 10285 subjects which suggested no CV harm with HR = 0.91 (95% CI: 0.68–1.22) [61]. Primary CV endpoints have just been made available with the CANagliflozin cardioVascular Assessment Study (CANVAS). In this trial, the integrated renal and CV pool of 10142 participants were reported together to maximize the power. Adults, above 50 years of age with established CV disease or above 60 years with two or more risk factors, received canagliflozin 100 mg or 300 mg or placebo in a 1 : 1 : 1 ratio, and they were followed over a 3.6-year period. The average age was 63.3 years, average BMI was 32.0 Kg/m^2^, and duration of T2D was 13.5 years. There was a significantly lower risk of the 3-point MACE in the canagliflozin group reported in absolute numbers as 26.9 versus 31.5 participants with an event per 1000 patient-years, conferring a HR = 0.86 (95% CI: 0.75 to 0.97; *p* = 0.02 for superiority); however, none of the three components were significant on their own. In contrast, hospitalization rate for heart failure was markedly lower in the canagliflozin group, with 5.5 versus 8.7 participants with an event per 1000 patient-years with HR of 0.67 (95% CI: 0.52–0.87) [62]. Furthermore, the benefit was observed within six months of entry into the trial and sustained throughout. Adverse events were lower overall in the canagliflozin group; however they were consistent with previous reports on SGLT2 inhibitors.

Dapagliflozin (FDA approved in 2014) was the first SGLT2 inhibitor to become available outside the USA. The initial submission to FDA in 2011 was refuted based on concern for bladder and breast cancer. However, after review of two additional years of data and an increase of 50% in patient-year exposure to dapagliflozin, an analysis of 11000 patients with T2D revealed reassuring CV safety profile [63]. A primary outcomes trial, the Dapagliflozin Effect on CardiovascuLAR Events-Thrombolysis in Myocardial Infarction 58 (DECLARE-TIMI 58), enrolled more than 17000 subjects in 2013, and results are anticipated for 2019 [64]. Until then, two published studies are in favor of dapagliflozin: a meta-analysis of the phase 2b/3 studies suggested no increase in mortality, nor in the 3-point MACE [65]. Even more favorably, a retrospective case-control open-cohort population-based study reviewed all-cause mortality and CV events in 22,124 patients with T2D on dapagliflozin (*n* = 4444) or not on SGLT2i (*n* = 17680) and found a significant decrease in all-cause mortality of 8.4 versus 17.2 incidence rate per 1000 person-years with adjusted relative risk 0.50 (95% CI 0.33–0.75) in the dapagliflozin group versus the control, respectively [66]. Additionally, the difference in mortality persisted in subgroup analysis when examining the low risk population.

Empagliflozin (FDA 2014) also has a completed primary CV outcomes trial. The EMPA-REG study randomized 7020 adults with high CV risk or disease to empagliflozin 10 mg or 25 mg or placebo in addition to standard of care [67]. The population was very similar to the previously described primary CV outcome trials, with average age 63 years, average BMI of 30 Kg/m^2^, and more than 50% subjects with duration of diabetes of more than 10 years (EMPA-REG). After a follow-up of 3.4 years, there was a significant decrease in the 3-point MACE occurring in 490 out of 4687 (10.5%) in the empagliflozin group versus 282 out of 2333 (12.1%) in placebo, HR of 0.86 (95% CI: 0.74–0.99; *p* = 0.04 for superiority). The effect was largely driven by death from CV cause. Hospitalization for heart failure occurred in 4.1% in the placebo group versus 2.7% in empagliflozin conferring 35% risk reduction. Both the CV mortality and heart failure benefits were observed as early as 6 months into the trial and were sustained [67]. Based on the trial results, the FDA has issued an additional approval for empagliflozin to reduce CV death in T2D in December 2016.

Possible explanations for the unanticipated early beneficial and powerful results were hemodynamic (increased natriuresis, decrease in blood pressure) and metabolic (decreased waist circumference and weight, HbA1c decrease of 0.4%) in nature [68]. However, similar changes seen with other agents did not yield the same benefits as observed with empagliflozin or canagliflozin. One suggested theory is that an increase in ketone levels may be beneficial to the myocardium, especially an ischemic myocardium, providing an alternative source of energy [69]. One additional SGLT2i molecule, ertugliflozin, is currently undergoing Cardiovascular Outcomes Following Ertugliflozin Treatment in Type 2 Diabetes Mellitus Participants with Vascular Disease (VERTIS CV) [70].

### 3.12. Insulin

The main side-effects of insulin—weight gain and hypoglycemia—have led to cautionary recommendations when used in patients with high risk of CV disease. Nonetheless, the beneficial effect of insulin on vascular prevention was demonstrated in the UKPDS whereby the group on intensive therapy had macrovascular benefit after 10 years of trial completion [20].

However, trials primarily designed to assess CV effect of insulin and aiming at similar glycemic control in both arms are scarce. The only existing placebo-controlled trial achieving this aim used insulin glargine in Outcome Reduction with Initial Glargine INtervention (ORIGIN) and randomized 12537 subjects (mean age 63.5 years) with existing or high risk CV disease and prediabetes (11.5%) or T2D to receive glargine versus standard of care. The mean duration of T2D and HbA1c were similar in both groups and were 5.5 years and 6.4%, respectively. By the end of the study, the HbA1c was 6.2% in the glargine group and 6.5% in the standard care, with more incidence of severe hypoglycemia occurring in the glargine group. After a 6.2-year follow-up, there were similar rates of 3-point MACE with 2.94 and 2.85 per 100 person-years, for glargine and standard care, respectively. There were 310 versus 343 hospitalizations for congestive heart failure in the glargine or standard care, respectively; however it did not reach significance with HR of 0.90 (95% CI: 0.77–1.05, *p* = 0.16) [71].

The DEVOTE trial (Comparing Cardiovascular Safety of Insulin Degludec versus Insulin Glargine in Patients with Type 2 Diabetes at High Risk of Cardiovascular Events) randomized 7637 with T2D at high CV risk to either glargine or degludec, for an average of 1.99 years. The mean age was 65.0 years, BMI was 33.0 Kg/m^2^, HbA1c was 8.4%, and duration of disease was 16.4 years. The results demonstrated noninferiority of degludec as compared to glargine, with respect to 3-point MACE. There were lower rates of hypoglycemia, including severe hypoglycemia [72]. Given that hypoglycemia represents an undesirable effect, especially in CV disease, it is worth noting that a more concentrated formulation of glargine U-300 was compared to glargine U-100 in both T1D and T2D and revealed less nocturnal hypoglycemia for the same level of glycemic control [73].

The Hyperglycemia and Its Effect after Acute Myocardial Infarction on Cardiovascular Outcomes in Patients with Type 2 Diabetes Mellitus (HEART2D) study was designed to target postprandial glucose with short-acting insulin as compared to fasting glucose with basal insulin in patients with acute MI occurring within 3 weeks of randomization [74]. After a 2.7-year follow-up on 1115 subjects, both groups achieved target HbA1c of 7% and the group on short-acting insulin had lower postmeal glucose excursions. However, there was no difference in the incidence neither of primary CV events nor on congestive heart failure.

Finally, a systematic review and meta-analysis comparing the CV outcomes of insulin versus noninsulin therapy included 18 trials and 5546 composite events occurring similarly in both arms [75]. It is important to note that only two out of these 18 trials extended beyond two years.

In brief, glucose-lowering with insulin provides macrovascular benefits, and limited studies have demonstrated its safety with respect to noninsulin therapy. The type and duration of action of insulin does not seem to affect the CV outcome; however it does impact on rates of hypoglycemia. Therefore, regimens should be judiciously prescribed to minimize this risk in patients with CV disease.

**Figure 1:**
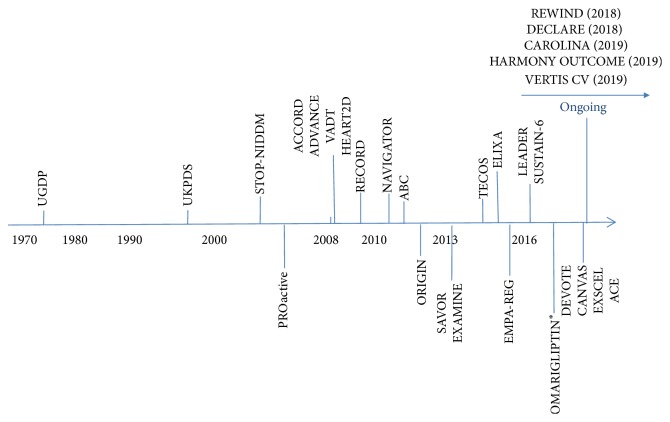
The landscape of cardiovascular trials in T2D. ^*∗*^Trial terminated.

Table 1 summarizes the primary outcome of the completed and reported trials of GLT and CV outcome, after the 2008 FDA mandate.  Figure 1 illustrates the timeline of the trials conducted in T2D with the primary outcome of CV disease.

## 4. Non-GLT Therapy with Hypoglycemic Benefits

Sacubitril belongs to a new class of drugs used for heart failure, the neprilysin inhibitors. Neprilysin is an enzyme expressed in the endothelium, cardiac myocytes, and adipocytes among other cells, responsible for the breakdown of a variety of vasoactive peptides such as natriuretic peptide, angiotensins I and II, bradykinin, and GLP-1. When combined with an Angiotensin Receptor Blocker (ARB), the sacubitril/valsartan net effect was shown to be favorable metabolically, with improved insulin sensitivity and glycemic control [76]. In the diabetes substudy of the PARADIGM-HF (Prospective Comparison of ARNI [Angiotensin Receptor-Neprilysin Inhibitor] with ACEI [Angiotensin-Converting-Enzyme Inhibitor]) to Determine Impact on Global Mortality and Morbidity in Heart Failure Trial, sacubitril/valsartan was compared with enalapril in 3778 subjects with New York Heart Association Classes II-IV heart failure and T2D. The combination in the original PARADIGM-HF study was overwhelmingly more powerful in reducing all-cause mortality, including death from CV cause, reducing hospitalization for heart failure, and improving physical symptomatology and functionality [77]. In addition, subjects with T2D had mildly lower HbA1c values (an absolute difference of −0.14%, 95% CI 0.06–0.23), needed to start insulin 29% less time than controls, and trended towards needing less oral hypoglycemic therapy [78].

The example of this new class of drugs, already FDA approved for the treatment of heart failure, blurs the margin between management of T2D and that of CV disease. These two conditions meet at many pathophysiological states and therefore ideally should be treated with drugs that have mutual beneficial effect.

## 5. Current Guidelines

The updated guidelines have taken into consideration recent evidence. Four points of agreement among the ones reviewed below are as follows:

(1) T2D management should be individualized with patient-specific glycemic targets.

(2) Lifestyle modification remains a mainstay therapy in the management.

(3) Metformin is the initial drug of choice.

(4) None of the guidelines reviewed incorporates the following 3 classes which are rarely used in T2D: bile acid sequestrants, amylin analogues, and dopamine agonists. Due to paucity of data, these will not be further discussed.

The Diabetes Australia 2016–2018 guidelines favor adding an SU, a DPP-4 inhibitor, or an SGLT2 inhibitor and if necessary, the addition of any of the above with GLP-1 agonists or insulin. The CV benefit observed with recent trials is mentioned, however not incorporated into a recommendation [79].

Diabetes Canada (previously called Canadian Diabetes Association) provided an interim update in November 2016 on glycemic management, whereby individuals are stratified according to the presence of CV disease or not. If present, then liraglutide or empagliflozin is to be considered next in line to metformin. If absent, then any of the classes of GLT would be suitable weighing in all the factors [80].

The National Institute for Health and Care Excellence (NICE) guidelines updated January 2017 mention as first intensification SUs, pioglitazone, or DPP-4 inhibitors, with certain restrictions on the use of pioglitazone [81]. The addition of SGLT2 inhibitor is mentioned in instances when SUs are not tolerated or hypoglycemia is significant. The addition of GLP-1 agonist is to be considered as a third line whenever BMI is above 35 kg/m^2^ or the use of insulin in those with a lower BMI would be restrictive occupationally. The CV outcomes observed with GLP-1 agonists and SGLT2 inhibitors are not incorporated into the algorithm.

The American Association of Clinical Endocrinologists (AACE) 2017 provides an algorithm with a hierarchical addition of GLT, with respect to the order of class suggested. After metformin, GLP-1 analogues and SGLT2 inhibitors would be second and third drugs, respectively. Whereas all classes are proposed as potential additions in case the SGLT2 inhibitors or GLP-1 analogues are not used, the guidelines point out that the use of TZDs, secretagogues, or insulin should proceed with caution. However, insulin is definitely recommended when triple therapy fails. Even though the guidelines emphasize patient individualization, they do no differentiate between CV risk or not when going through the hierarchy [82].

In contrast, the ADA in January 2017 incorporated new evidence as follows: after metformin all classes of drugs are possible second option if there is no increased CV risk. Even though they are provided as choices, meglitinides and AGis are not incorporated into the algorithm. Furthermore, if CV disease is established, then empagliflozin and liraglutide are to be considered due to the demonstration of benefit [1].

Finally, in terms of cardiology societies, the American Heart Association's last update on prevention of CV disease in adults with T2D was in 2015, jointly with the ADA. After metformin, the guidelines favor adding pioglitazone and acarbose. However, they were formulated prior to the most recent positive studies and therefore require an update before they can be followed [83]. The European Society of Cardiology (ESC May 2016) guidelines on chronic and acute heart failure do mention empagliflozin favorably after metformin and caution regarding insulin, TZDs, and secretagogues [84].

The new results of sacubitril/valsartan trial and GLP-1 RA and SGLT2i studies will likely shape the future management of T2D in CV disease, and upcoming society guidelines will likely be even more closely intertwined and multidisciplinary in nature.

## 6. Summary

**Figure 2:**
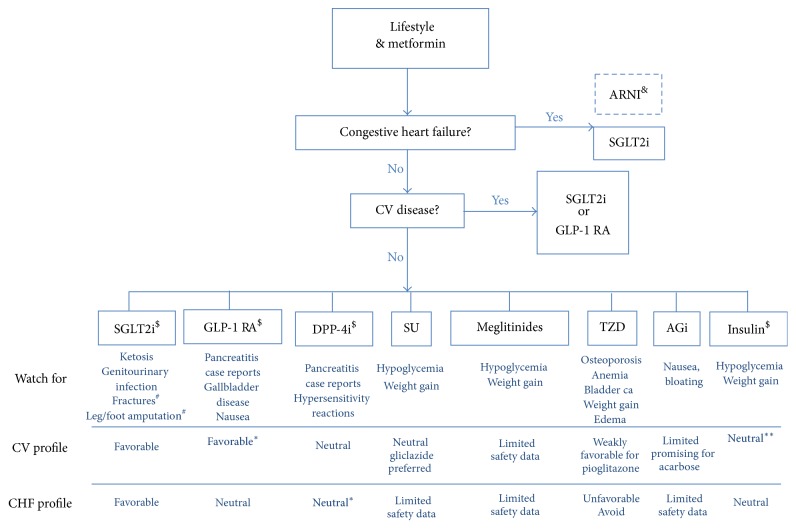
Suggested algorithm for pharmacotherapy in T2D. ^*∗*^Agent-specific. ^*∗∗*^Grade A evidence with glargine and degludec. ^#^Effect reported for canagliflozin. ^&^Not among the FDA-approved classes of GLT, however potential advantage in heart failure. ^$^Cost may be a significant consideration. SGLT2i: sodium-glucose cotransport 2 inhibitor; GLP-1 RA: glucagonlike peptide-1 receptor agonist; DPP-4 i: dipeptidyl peptidase-4 inhibitor; SU: sulfonylurea; TZD: thiazolidinedione; AGi: alpha glucosidase inhibitor; ARNI: Angiotensin Receptor blocker Neprilysin Inhibitor.

From our own synthesis of the trial findings, we propose the following steps, as shown in Figure 2: firstly, lifestyle recommendation and the addition of metformin should remain the first step and the backbone of management of T2D; secondly, one needs to assess for cardiac risk; in case heart failure is present, then SGLT2i is added preferentially after metformin, followed by GLP-1 RA. We make a footnote regarding the glycemic benefit of neprilysin inhibitors/ARB combination, without recommending its addition for glycemic control primarily at this point. In case there is no heart failure risk but there is concern for atherosclerotic disease, then either SGLT2i or GLP-1 RA is recommended after metformin. Thirdly, if additional glucose-lowering is required, then gliclazide, pioglitazone, DPP-4 inhibitors, and basal insulin would be favored options. The upcoming ACE trial results, if positive, may also propel acarbose into an equally viable option. As a fourth step, if neither heart failure nor CV risk is present, then glycemic control can be achieved with any of the classes mentioned above, with special attention paid to patient risk factors and knowledge of side-effect profile such as risk of ketosis, pancreatitis, cholecystitis, osteoporosis, genital infections, and bladder cancer. Cost, feasibility, risk of hypoglycemia, and long-term glycemic control are factors to be considered (Figure 2). Cost consideration, in particular, would apply to the newer agents such as SGLT2i, DPP-4i, GLP-1 RA, and the “designer” insulin.

The Glycemia Reduction Approaches in Diabetes: A Comparative Effectiveness Study (GRADE) is an ongoing trial of 4–7 years aimed at comparing SU, insulin, DPP-4 inhibitor, and GLP-1 agonist for glycemic control and durability, which should help shed further light into the algorithm stratification [85]. In addition, whether the benefit of a drug is a class effect or a molecule effect remains to be proven within the next few years. To make the conclusion more complex, it is very difficult to make evidence-based recommendations regarding combinations. For example, despite the individual benefit of GLP-1 RA and SGLT2i, one cannot conclude that their combination will yield the same benefit. If glucagon is central to the advantage observed with SGLT2 inhibitors, then lowering it with GLP-1 may be detrimental. Only trials with the combination may be able to address this point. One such trial using once-weekly exenatide and dapagliflozin did show beneficial metabolic endpoints after 28 weeks of use [86]. However, the cost of such study of combination and CV assessment may be prohibitive. So, despite the accumulating evidence, the fine-tuning of glycemic control will always draw on the “art” of medicine, as well as its science. Last but not least, CV prevention in T2D is multifactorial and attention should be paid to lipids, smoking cessation, blood pressure control, obesity, and albuminuria. The benefit of multifactorial intervention was shown again by the extension of the STENO study, whereby 7.8 years of metabolic control increased lifespan by 7.9 years and delayed CV events by 8.1 years [87].

## 7. Concluding Remarks

The amount of knowledge over the past decade on glucose-lowering and CV effects has improved significantly. The choice of glucose-lowering medication has widened and remains patient-centered based on risk profile and potential benefit. The current review of the CV outcomes of all the available drugs with a perspective on the historical evolution of diabetes therapy keeps the available classes in context. The choice of drugs is likely to evolve further with refinement of our current knowledge. There were great strides made since the 10-year follow-up on the UKPDS showing CV risk reduction with improved glycemia. Therefore, addressing pharmacotherapy of T2D judiciously as highlighted in this paper should be carried out as part and parcel of overall patient well-being.

## Figures and Tables

**Table 1:** Summary of the completed trials of hypoglycemic therapy and cardiovascular outcome as a result of the 2008 FDA mandate.

Trial	Tested molecule	Comparator	Population	*N*	Duration (years)	Primary outcome	Hazard ratio
ORIGIN [71]	Glargine	Standard care	Prediabetes or T2D with CV risk	12537	6.2	3-point MACE	1.02 (0.94–1.11)
DEVOTE [72]	Degludec	Glargine	T2D and above 50 years with CV or renal, or above 60 years at risk	7637	2.0	3-point MACE	0.91 (0.78 to 1.06)
SAVOR [41]	Saxagliptin	Placebo	T2D and above 40 with CV disease or above 55 years at risk	16492	2.1	3-point MACE	1.00 (0.89–1.12)
EXAMINE [43]	Alogliptin	Placebo	T2D and acute coronary syndrome within 15-90 days	5380	1.5	3-point MACE	0.96 (≤1.16)
TECOS [44]	Sitagliptin	Placebo	T2D and above 50 years and established CV disease	14671	3.0	4-point MACE	0.98 (0.88–1.09)
ELIXA [56]	Lixisenatide	Placebo	T2D and patients with acute coronary syndrome within 180 days	6068	2.1	3- point MACE	1.02 (0.89–1.17)
EMPA-REG [67]	Empagliflozin	Placebo	T2D and age above 18 years with established CV disease	7020	3.1	3-point MACE	0.86 (0.74–0.99)
CANVAS [62]	Canagliflozin	Placebo	T2D and age above 30 years with CV or above 50 years with ≥ 2 risks	10142	3.6	3- point MACE	0.86 (0.75 to 0.97)
LEADER [57]	Liraglutide	Placebo	T2D and age above 50 years and CV disease or above 60 years at risk	9340	3.8	3-point MACE	0.87 (0.78–0.97)
SUSTAIN-6 [58]	Semaglutide	Placebo	T2D and above 50 years with CV disease or age above 60 years at risk	3297	2.1	3- point MACE	0.86 (0.74–0.99)
ACE [88]	Acarbose	Placebo	IGT and above 50 years with CV disease	6522	5.0	5-point MACE	0.98 (0.86–1.11)
EXSCEL [89]	Exenatide	Placebo	T2D adults with established CV disease 70% or at risk 30%	14752	3.2	3-point MACE	0.91 (0.83–1.00)

*Notes*. Overall, study population across studies represents a high cardiovascular risk, with average age ranging from 60 to 65 years (±SD 7–10 years), mean BMI range is from 28.7 to 32.8 kg/m^2^, with diabetes duration ranging between 5.4 and 13.8 years, and average HbA1C range is from 6.4 to 8.7% (±0.8–1.5%). Table adapted from Table 1 from [2].

## Nomenclature

### Abbreviations

AACE: American Association of Clinical Endocrinologists
ADA: American Diabetes Association
AGi: Alpha glucosidase inhibitor
AHA: American Heart Association
ARB: Angiotensin Receptor Blocker
ARNI: Angiotensin Receptor Blocker Neprilysin Inhibitor
BMI: Body Mass Index
CI: Confidence interval
CV: Cardiovascular
DPP-4i: Dipeptidyl peptidase-4 inhibitor
FDA: Food and Drug Administration
GLP-1 RA: Glucagonlike peptide-1 receptor agonist
GLT: Glucose-lowering therapy
HbA1c: Hemoglobin A1c
MACE: Major Adverse Cardiovascular Events
MI: Myocardial infarction
NICE: National Institute for Health and Care Excellence
SGLT2i: Sodium-glucose cotransport 2 inhibitor
SU: Sulfonylurea
T2D: Type 2 Diabetes
TZD: Thiazolidinedione.

#### Trial Acronyms

ABC: Alpha-glucosidase-inhibitor Blocks Cardiac Events in Patients with Myocardial Infarction and Impaired Glucose Tolerance
ACE: Acarbose Cardiovascular Evaluation
ACCORD: Action to Control Cardiovascular Risk in Diabetes
ADVANCE: Action in Diabetes and Vascular Disease: Preterax and Diamicron MR Controlled Evaluation
CANVAS: CANagliflozin cardioVascular Assessment Study
CAROLINA: CARdiovascular Outcome study of LINAgliptin versus glimepiride in patients with Type 2 Diabetes
DECLARE-TIMI 58: Dapagliflozin Effect on CardiovascuLAR Events-Thrombolysis in Myocardial Infarction 58
DEVOTE: Comparing Cardiovascular Safety of Insulin Degludec versus Insulin Glargine in Patients with Type 2 Diabetes at High Risk of Cardiovascular Events
ELIXA: Evaluation of LIXisenatide in Acute coronary syndrome
EMPA-REG: Empagliflozin, Cardiovascular Outcomes, and Mortality in Type 2 Diabetes
EXAMINE: Examination of Cardiovascular Outcomes: Alogliptin versus Standard of Care in Patients with Type 2 Diabetes Mellitus and Acute Coronary Syndrome
EXSCEL: Exenatide Study of Cardiovascular Event Lowering
GRADE: Glycemia Reduction Approaches in Diabetes: A Comparative Effectiveness Study
HARMONY OUTCOME: Effect of Albiglutide, When Added to Standard Blood Glucose-Lowering Therapies, on Major Cardiovascular Events in Subjects with Type 2 Diabetes Mellitus
HEART2D: Hyperglycemia and Its Effect after Acute Myocardial Infarction on Cardiovascular Outcomes in Patients with Type 2 Diabetes Mellitus
LEADER: Liraglutide Effect and Action in Diabetes: Evaluation of Cardiovascular Outcome Results
NAVIGATOR: Long-Term Study of Nateglinide + Valsartan to Prevent or Delay Type II Diabetes Mellitus and Cardiovascular Complications
ORIGIN: Outcome Reduction with Initial Glargine INtervention
PARADIGM-HF: Prospective Comparison of ARNI [Angiotensin Receptor-Neprilysin Inhibitor] with ACEI [Angiotensin-Converting-Enzyme Inhibitor]
PROactive: PROspective PioglitAzone Clinical Trial in macroVascular Events
RECORD: Rosiglitazone Evaluated for Cardiovascular Outcomes in oRal agent combination therapy for type 2 Diabetes
REWIND: Researching Cardiovascular Events with a Weekly Incretin in Diabetes
SAVOR-TIMI 53: Saxagliptin Assessment of Vascular Outcomes Recorded in patients with diabetes mellitus-Thrombolysis in Myocardial Infarction 53
STENO-2: Intensified multifactorial intervention and cardiovascular outcome in type 2 diabetes
STOP-NIDDM: Stop Non-Insulin Dependent Diabetes Mellitus
SUSTAIN-6: Trial to Evaluate Cardiovascular and Other Long-Term Outcomes with Semaglutide in Subjects with Type 2 Diabetes
TECOS: Trial Evaluating Cardiovascular Outcomes with Sitagliptin
UGDP: University Group Diabetes Program
UKPDS: United Kingdom Prospective Diabetes Study
VADT: Veterans Affairs Diabetes Trial
VERTIS CV: Cardiovascular Outcomes Following Ertugliflozin Treatment in Type 2 Diabetes Mellitus Participants with Vascular Disease
VIVIDD: Vildagliptin in Ventricular Dysfunction Diabetes.

## Additional Points

*Postscript*. After acceptance of the paper, two trials which were listed as pending were actually presented and published September 13 and 14, 2017, for ACE and EXSCEL, respectively [88, 89]. The ACE trial conducted on 6522 Chinese adults above age of 50 years with known CV disease and impaired glucose tolerance was started in 2009 and completed in April 2017. The trial used an intermediate dose of acarbose of 50 mg three times daily, and it had a higher adherence rate of above 96% as compared to STOP-NIDDM. The results, however, were minimal for metabolic improvement in favor of acarbose, and the primary outcome (5-point MACE) showed no difference between the two groups (HR = 0.98; 95% CI: 0.86–1.11). The second aim of the study which was diabetes prevention did show an 18% reduction in the risk of T2D, a fact already known for this class [88]. The EXSCEL trial was an event-driven trial which reached its target earlier than anticipated. This study was conducted on 14,752 adults with known CV disease (70%) or at high risk (30%) and T2D who were given once-weekly exenatide of 2 mg or placebo, with the standard of care, and they were followed for a median of 3.2 years. There was no difference in the 3-point MACE (HR = 0.91; 95% CI: 0.83–1.00, *p* < 0.001 for noninferiority). However, all-cause mortality was slightly lower in the exenatide group versus placebo group (HR = 0.86; 95% CI: 0.77–0.97). There was no progression to retinopathy and the drug was well-tolerated overall [89].
